# Deciphering the mitochondrial genome of *Hemerocallis citrina* (Asphodelaceae) using a combined assembly and comparative genomic strategy

**DOI:** 10.3389/fpls.2022.1051221

**Published:** 2022-11-18

**Authors:** Kun Zhang, Yiheng Wang, Xun Zhang, Zhiping Han, Xiaofei Shan

**Affiliations:** ^1^ College of Agriculture and Life Sciences, Shanxi Datong University, Datong, Shanxi, China; ^2^ Institute of Germplasm Resources and Biotechnology, Tianjin Academy of Agricultural Sciences, Tianjin, China

**Keywords:** *Hemerocallis citrina*, Asphodelaceae, mitochondrial genome, combined sequencing, comparative genomics

## Abstract

*Hemerocallis citrina* is a perennial herbaceous plant that is dedicated to mothers in Chinese culture and is widely distributed across the country. As a popular species with a long history of cultivation and utilization, it is renowned for its remarkable edible and medicinal value. In this study, we integrated Illumina short-read and Oxford Nanopore long-read sequencing to generate a complete mitochondrial genome (mitogenome) assembly of *H. citrina.* The *H. citrina* mitogenome has a multiple chromosomal structure consisting of three circular molecules that are 45,607 bp, 239,991 bp, and 182,864 bp long. We correspondingly annotated 66 genes, comprising 45 protein-coding genes (PCGs), 17 tRNA genes, and 4 rRNA genes. Comparative analysis of gene organization indicated that six syntenic gene clusters were conserved in the mitogenomes of the compared plants. The investigation of repeat content revealed repeat-rich nature of the *H. citrina* mitogenome, for which plentiful dispersed repeats were characterized to correlate with the size of the mitogenome. The codon usage behavior disclosed that Leucine (Leu) and Serine (Ser) were the most preferred amino acids in *H. citrina*, and nearly all of the codons with relative synonymous codon usage (RSCU) values greater than 1 showed the preference of A or T ending. Moreover, we inferred a total of 679 RNA editing sites in all mitochondrial PCGs, which presented perfect C-to-U types and tended to lead to the alteration of internal codons. Subsequent selective pressure analysis showed that the majority of the PCGs had undergone evolutionary negative selections, with *atp9* in particular undergoing strong stabilizing selection, reflecting its indispensable function in mitogenomes. According to the phylogenetic analysis, *H. citrina* is close to the species *Allium cepa* (Amaryllidaceae) and *Asparagus officinalis* (Asparagaceae) in evolutionary terms. Overall, this project presents the first complete mitogenome of *H. citrina*, which could provide a reference genome for the comprehensive exploration of the Asphodelaceae family and can facilitate further genomic breeding and evolutionary research on this medicine–food homologous plant.

## Introduction

Mitochondria (mt) are critical organelles that are ubiquitous in eukaryotic cells and drive the energy metabolism for biological growth, development, and reproductive processes (Liberatore et al., 2016). They retain their own genomes that are inherited independently from nuclear genomes through ancient endosymbiotic events (Timmis et al., 2004). With the ongoing advancements in genome sequencing and assembly technologies, there has been rapid progress in deciphering plant mt genomes (mitogenomes). In contrast to conserved plastid genomes, plant mitogenomes exhibit more complex genome structures, broad genome size variations, numerous RNA editing modifications, abundant repeated sequences, sparse gene distribution, and continual gene relocation during evolution (Handa, 2003; Kubo and Mikami, 2007; Alverson et al., 2010; Sloan et al., 2012; Zhang et al., 2019; Kan et al., 2020). These unique features complicate the sequencing and annotation of plant mitogenomes. Although more than 350 complete mitogenomes of land plants are available in the NCBI (National Center for Biotechnology Information, https://www.ncbi.nlm.nih.gov/genome/browse#!/organelles/., 18/07/2022), this number is miniscule compared to the complete genome data for thousands of plant chloroplasts (cp) and animal mt.

The evolution of plant mitogenomes has involved complex genomic rearrangements, contributing to tremendous variations in the size and structure of mitogenomes across different species (Wu et al., 2020). Current known sizes range from the standard dozens and hundreds of kilobases to a few notable exceptions that are larger than one megabase (Sloan et al., 2012; Skippington et al., 2015; Jackman et al., 2020; Li et al., 2021a). As already researched and documented, mitogenome size can fluctuate greatly, even among species that are closely related in evolutionary terms (Alverson et al., 2010). The divergence of mitogenome size is principally driven by the accumulation of noncoding regions, consisting of dispersed repeats, introns, intergenic segments, and the integration of foreign DNA, rather than by differences in gene content (Allen et al., 2007; Alverson et al., 2011; Goremykin et al., 2012; Rice et al., 2013). Thus, plant mitogenomes present comparative conservation in functional genes, and in particular, genes associated with mt respiration and energy synthesis are deemed to be highly conserved in most plant mitogenomes. However, there is also evidence that *Viscum scurruloideum* has lost all *nad* genes contributing to respiratory Complex I (NADH dehydrogenase) (Skippington et al., 2015), resulting in diverse and complex plant mitogenomes in terms of the gene content. In addition to their size and gene category, plant mitogenomes have also been found to possess multiple structures. The majority of plant mitogenomes established so far can be portrayed as single circular chromosomes, which were once assumed to be the dominant form of the mt structure. However, subsequent studies confirmed that the actual architecture of the mitogenome appears to occur in circular, linear, and complex branched arrangements (Backert and Börner, 2000; Smith and Keeling, 2015). These variable features render the plant mitogenome an attractive genetic system for the study of issues relevant to evolutionary biology, as well as laborious to assemble and annotate. Nevertheless, plant mitogenomes remain somewhat mysterious because of limited sampling, which hinders an overall understanding of their diversity and evolution.

*Hemerocallis citrina* Borani, a popular perennial herbaceous plant mainly distributed in China, belongs to the family Asphodelaceae*. H. citrina*, also known as “Daylily” or “Huang hua cai”, has been respected as the “mother flower” in traditional Chinese culture for thousands of years (Ma et al., 2018; Liu et al., 2020). The immature flower buds are commonly processed into a dried vegetable and consumed as a nutraceutical food in Asia. Due to its profusely high flavonoid and polyphenol contents (Xu et al., 2016), *H. citrina* has the concomitant function of both a medicine and a foodstuff. *H. citrina* has been proposed as a Chinese herbal medicine in antioxidant, anti-inflammatory, and antidepressant treatments for a long time. Research on *H. citrina* has mainly focused on morphological assessment (Li et al., 2021b), pharmaceutical properties (Wang et al., 2018), tissue culture (Matand et al., 2020), and circadian flowering rhythm (Ren et al., 2019). In recent years, sequencing information of the cp (Ou et al., 2020) and nuclear (Qing et al., 2021) genomes in *H. citrina* has been successively obtained and utilized. However, further knowledge of the mt genome is urgently needed for the evolutionary understanding and genetic improvement of *H. citrina.*

In this study, we sequenced and completed the first *H. citrina* mitogenome from Asphodelaceae utilizing the Illumina short-read and Nanopore long-read integrated pipeline. The assembled *H. citrina* genome was characterized for gene annotation, sequence variation, selection pressure, RNA editing events, and phylogenetic position by comparing its counterparts with published plant mitogenomes. This information on the *H. citrina* mitogenome will fill the mitogenomic evolution knowledge gap for the *Hemerocallis* genus and will further facilitate genetic and evolutionary studies of this extremely valuable plant.

## Materials and methods

### Plant materials and genome sequencing

For DNA extraction, fresh leaves of *H. citrina* (cultivar *Datong hua*) were collected from Datong, Shanxi Province, China. High-quality genomic DNA was isolated employing a modified CTAB protocol (Porebski et al., 1997). The purity, concentration, and integrity of the extracted DNA were examined using a NanoDrop (Thermo Scientific, USA), a Qubit fluorometer (Thermo Scientific, USA), and 0.35% agarose gel electrophoresis, respectively. Both short-read (Illumina) and long-read (Oxford Nanopore) sequencing technologies were implemented to obtain full-length mitogenome sequences. To collect large DNA sequences, optional fragmentation was performed using the BluePippin system (Sage Science, USA). Subsequently, the fragmented DNA was treated using damage repair, end preparation, A-tailing, and adapter ligation and then cleaned with magnetic beads. A library > 8 kb was constructed following the SQK-LSK109 (Oxford Nanopore Technologies, ONT, UK) sequencing kit protocol and loaded into a Nanopore GridION Sequencer (ONT, UK).

### Genome assembly and annotation

This whole genome sequencing produced 12.6 Gb in 1.3 million long-reads (SRA accession SRR15370204). The raw long-reads were filtered and re-edited using NanoFilt and NanoPlot in Nanopack (De Coster et al., 2018), then a total of 11.6 Gb clean data were generated. Meanwhile, libraries with an average fragment length of 350 bp were prepared utilizing the NEBNext^®^ Ultra™ DNA Library Prep Kit (New England Biolabs, England) and then sequenced on the Illumina Novaseq 6000 platform (Illumina, USA) with 150 bp paired-end reads. The Illumina short-read sequencing yielded 10.2 Gb in 34.1 million reads (SRA accession SRR15370205). The quality of the raw short-reads was assessed using the NGS QC Tool Kit v2.3.3 (Patel and Jain, 2012). A total of 10.1 Gb clean short-reads were obtained by removing adapter sequences and low-quality reads using Cutadapt (v1.9.1). First, we obtained a rough but computationally efficient assembly *via* Miniasm (Li, 2016) after trimming adapter sequences with Porechop v0.2.4 (Wick et al., 2017) and polishing the resulting assembly with Racon v1.4.7 (Vaser et al., 2017). Subsequently, potential mt contigs with homology to the *Asparagus officinalis* mitogenome (NCBI Reference Sequence: MT483944.1) were obtained using Bandage (Wick et al., 2015). We then proceeded to align the Nanopore reads to our draft *H. citrina* assembly using minimap2 (Li, 2018) and segregated aligned reads and reassembled them, first using Flye v2.6 (Kolmogorov et al., 2019), and then using Canu v2.1.1 (Koren et al., 2017). The final genome sequence was obtained by polishing with Pilon (Walker et al., 2014) using Illumina Novaseq sequencing reads. Based on the above assembly procedures, we obtained a three-ring structure of the *H. citrina* mitogenome.

The complete mitogenome of *H. citrina* was annotated using MITOFY (Alverson et al., 2010) and MFANNOT (Gautheret and Lambert, 2001), utilizing previous angiosperm mt genes to query sequences in the NCBI database (https://www.blast.ncbi.nlm.nih.gov). The tRNA genes were detected using tRNAscan-SE 2.0 (Schattner et al., 2005). The chloroplasts (cp) genome of *H. citrina* (MN872235.1) was used to determine the tRNA genes transferred from cp to mt. The origins of tRNA genes were identified using BLASTn on tRNAscan-SE 2.0 with the matching rate set to ≥ 70%, length ≥ 30, and E-value ≤ 1e^-3^. The relative synonymous codon usage (RSCU) values and amino acid composition of PCGs were calculated using CodonW (http://codonw.sourceforge.net/). The circular and syntenic gene cluster maps of the *H. citrina* mitogenome were visualized using the OGDRAW program (Greiner et al., 2019). The assembled complete mitogenome sequence of *H. citrina* has been submitted to GenBank (NCBI) and is openly available under accession numbers: MZ726801.1, MZ726802.1, and MZ726803.1.

### Genome analysis

To identify mitochondrial synteny blocks of the *H. citrina* compared with representative species, 7 pairs of monocotyledonous mitogenomes were aligned using Mauve v2.4.0 with a LCB cutoff of 42 (Darling et al., 2010). The amount of shared mtDNA was determined using BLASTn with the parameters word_size 7 and e-value 1×10^−6^. Three types of repeat sequences were identified in the *H. citrina* mitogenome. Simple sequence repeats (SSRs) were searched using MISA-web (https://webblast.ipk-gatersleben.de/misa/) (Beier et al., 2017) with preset thresholds of eight, four, four, three, three, and three corresponding to the motif size of one to six nucleotides, respectively. The Finder 4.09 program (http://tandem.bu.edu/trf/trf.html) was utilized to confirm and locate tandem repeats using default parameters (Benson, 1999). Dispersed repeats were identified using the public tool REPuter (https://bibiserv.cebitec.uni-bielefeld.de/reputer) (Kurtz et al., 2001) with the repeat size set to > 30 bp. To infer the selection pressure of PCGs during the evolution of *H. citrina*, the nonsynonymous (Ka) and synonymous (Ks) substitution rates of each PCG were calculated for *H. citrina* and five other species (*Allium cepa*, *A. officinalis*, *Arabidopsis thaliana*, *Nelumbo nucifera*, and *Oryza sativa*). ParaAT2.0 was used with default settings to align and format the PCGs (Zhang et al., 2012). The calculations of the Ka, Ks, and Ka/Ks values were performed using KaKs_Calculator v.2.0 following the YN method (Wang et al., 2010). Putative RNA editing sites in the PCGs of *H. citrina* and the five other species were projected using PREP-Mt (http://prep.unl.edu/). To detect more true edits, the cutoff parameter was set to 0.2 (Mower, 2009).

### Phylogenetic tree construction

Nine conserved mt PCGs (*atp1*, *ccmB*, *ccmC*, *cob*, *nad3*, *nad4*, *nad4L*, *nad7*, and *nad9*) were lined up to construct a contiguous sequence and were then individually aligned using MAFFT 7.017 (Katoh and Standley, 2013) among the 29 species analyzed. The NCBI accession numbers of all these observed mitogenomes are listed in **Supplementary Table 1**. After ModelFinder was used to select the most appropriate amino acid substitution pattern, the maximum likelihood (ML) phylogenetic tree was established based on a GTR+F+R3 model using IQTREE v1.6 (Trifinopoulos et al., 2016) with 1000 bootstraps and *Ginkgo biloba* as an outgroup.

## Results

### Mitogenome assembly and genomic features

**Figure 1:**
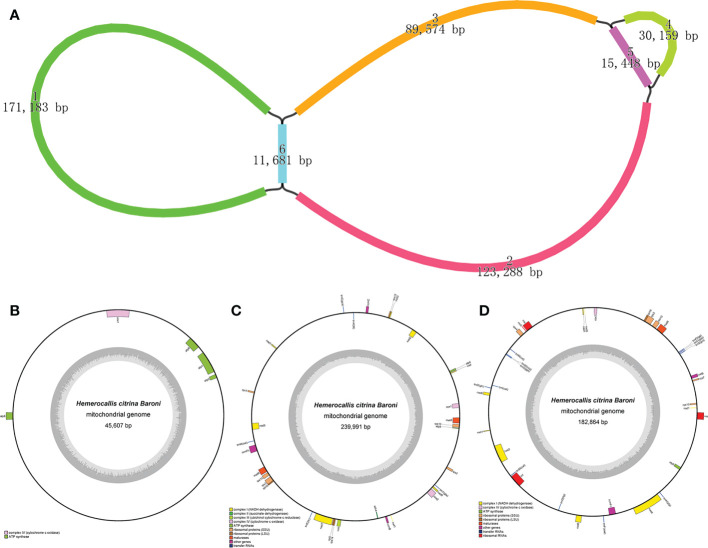
Assembly results of the *H. citrina* mitogenome. **(A)** The assembly graph of the *H. citrina* mitogenome. Each colored segment is labeled with its size and named 1-6 by rank of size. **(B-D)** Circular map of the *H. citrina* mitogenome. Genes presented on the outer circle are transcribed, whereas genes on the inside are counterclockwise. The inner circle reveals the GC content as a dark gray plot with the gray line in the middle as the 50% threshold line. **(B)** Schematic diagram of molecule 1 with a length of 45,607 bp and five annotated genes; **(C, D)** Similar maps for molecule 2 and molecule 3 with lengths of 239,991 bp and 182,864 bp, respectively. Because the three genes (*nad1, nad2*, and *nad5*) encoding the subunits of Complex I (NADH dehydrogenase) are fragmented in molecules 2 and 3, the two mt circular molecules encode a total of 61 genes.

As visualized in **Figure 1A**, we obtained six segments to construct the completed draft mt graph for assembling the *H. citrina* mitogenome. All segment adjacencies are supported by the long reads, suggesting that several combinations exist in the mitogenome of *H. citrina*. Since the three loops are the smallest indivisible units, the *H. citrina* mitogenome was assembled into three complete circular molecules that were 45,607 bp (molecule 1), 239,991 bp (molecule 2), and 182,864 bp (molecule 3) long (**Figures 1B-D**, **Supplementary Table 2**). The three molecules of the mitogenomes had an overall GC content of 44.4%, 45.1%, and 45.7%, respectively (**Table 1**). Notably, the lengths of the coding regions, without exception, occupied only a small part of the three mitogenomes.

**Table 1:** Genomic features of the *H. citrina* mitogenome.

Genome	Feature	A %	C %	G %	T %	GC %	Size (bp)	Proportion in Genome (%)
Molecule 1	Whole genome	27.9	22.4	22.0	27.7	44.4	45,607	100
	Protein-coding genes^a^	26.5	21.3	21.9	30.3	43.2	4,683	10.3
	*cis*-spliced introns^a^	–	–	–	–	–	–	–
	tRNA genes^a^	–	–	–	–	–	–	–
	rRNA genes^a^	–	–	–	–	–	–	–
	Non-coding regions	27.7	22.4	22.1	27.8	44.5	40,924	89.7
Molecule 2	Whole genome	27.4	22.5	22.5	27.6	45.1	239,991	100
	Protein-coding genes^a^	26.7	21.9	22.3	29.1	44.2	21,604	9.0
	*cis*-spliced introns^a^	23.4	26.1	27.6	22.9	53.7	11,094	4.6
	tRNA genes^a^	23.7	22.0	26.6	27.7	48.6	372	0.2
	rRNA genes^a^	–	–	–	–	–	–	–
	Non-coding regions	27.5	22.4	22.3	27.8	44.7	206,949	86.2
Molecule 3	Whole genome	27.4	22.7	23.0	26.9	45.7	182,864	100
	Protein-coding genes^a^	26.3	22.3	21.8	27.5	44.2	14,495	7.9
	*cis*-spliced introns^a^	25.3	24.1	26.9	23.7	51.0	12,046	6.6
	tRNA genes^a^	24.1	21.5	27.0	27.4	48.5	910	0.5
	rRNA genes^a^	25.4	23.2	30.0	21.3	53.3	7300	4.0
	Non-coding regions	27.6	22.5	22.5	27.4	45.0	148,142	81.0

a Protein-coding genes, cis-spliced introns, tRNAs, and rRNAs belong to coding regions.

-, absent.

**Figure 2:**
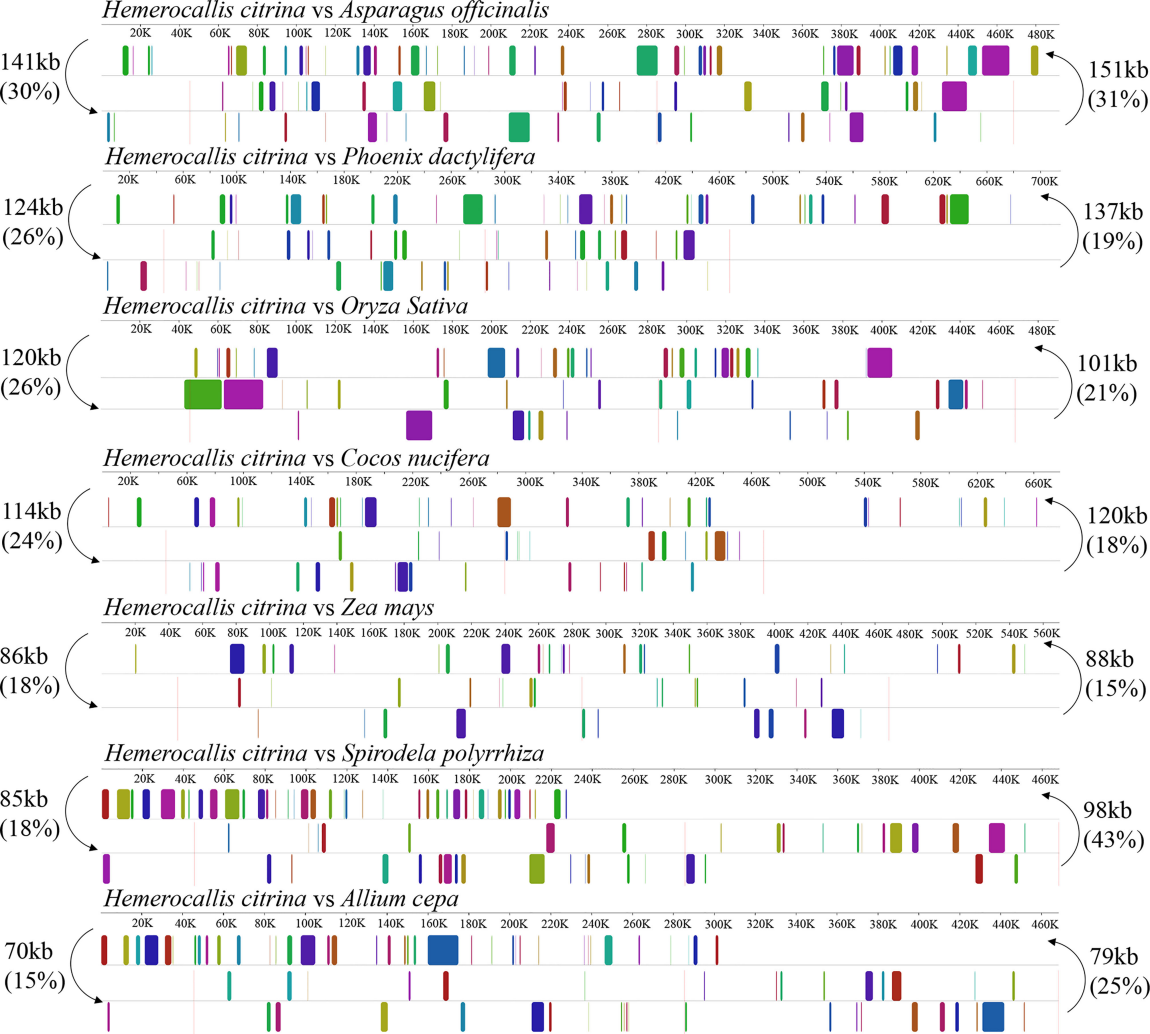
Synteny blocks and shared mtDNA of the *H. citrina* and seven other monocotyledons based on Mauve alignments. Left arrows indicate the amount of shared mtDNA, in kb and percentage, of the *H. citrina* and one other species, and right arrows present the reciprocal values.

To investigate the genome rearrangements in *H. citrina*, the quantity of shared DNA was measured between the *H. citrina* and the other 7 monocotyledons. As shown in **Figure 2**, the mitogenome sequence of *H. citrina* exhibited a low level of synteny and shared DNA compared with the other monocotyledons. For instance, only 30% (141 kb) of the 468 kb mitogenome of *H. citrina* is homologous to the *A. officinalis* mitogenome. Lower amounts of DNA are shared with the 26% (124 kb) of the shared DNA between *H. citrina* and *Phoenix dactylifera*, 26% (120 kb) shared between *H. citrina* and *O. sativa*, and 24% (114 kb) shared between *H. citrina* and *Cocos nucifera.* In stark contrast, the *H. citrina* and *A. cepa* mitogenomes retain merely 70 kb of shared DNA.

### Gene annotation and organization

The *H. citrina* mitogenome contains 66 annotated genes, representing 53 unique genes (**Table 2**). Forty-five protein-coding genes (PCGs) responsible for electron transport, oxidative phosphorylation, cytochrome c biogenesis, ATP synthesis, and ribosomal protein were identified in the mitogenome. PCGs comprised most of the coding regions, whereas their GC content was the smallest compared with other regions (**Table 1**). The genome includes 17 tRNA genes, of which 13 and 4 genes were of mt and cp origin, respectively. In addition, four rRNA genes were also found in *H. citrina.* Introns emerged in seven of the annotated genes, namely, *nad1*, *nad2*, *nad5*, *nad7*, *cox2*, *ccmFc*, and *rps10.* Additionally, three genes (*nad1*, *nad2*, and *nad5*) encoding subunits of Complex I were fragmented in molecules 2 and 3.

**Table 2:** Gene annotation of the *H. citrina* mitogenome.

Group of Genes	Gene Name			
	Molecule 1	Molecule 2	Molecule 3	Numbers
Complex I (NADH dehydrogenase)		*nad1^#^, nad2^#^, nad3, nad4L, nad5^#^, nad7^#^, nad9*	*nad1^#^, nad2^#^, nad4, nad5^#^, nad6*	9
Complex II (succinate dehydrogenase)		*sdh4*		1
Complex III (ubiquinol cytochrome c reductase)		*cob*		1
Complex IV (cytochrome c oxidase)	*cox1*	*cox1, cox2^#^*	*cox3*	4
Complex V (ATP synthase)	atp1, atp8(2)*, atp9	*atp4, atp9*	*atp6*	7
Cytochrome c biogenesis		*ccmB, ccmC, ccmFc^#^*	*ccmFn*	4
Ribosomal protein		rpl5, rpl16, rps1, rps2, rps3, rps10^#^(2)^*^, rps12, rps14	*rpl16, rps3, rps4, rps7, rps10^#^, rps13*	15
Ribosomal RNA			rrn5, rns(2)*, rnl	4
Transfer RNA		*trnC, trnE, trnK, trnM-cp, trnY*	trnD, trnF(2)*, trnH-cp, trnI, trnM, trnM-cp, trnP, trnQ, trnS(2)*, trnW-cp	17
Others		matR(2)*	*matR, mttB*	4
Total	5		61	66

Multicopy genes are presented with *, and bracketed numbers represent copy number of each gene. Genes with introns are denoted with ^#^.

**Figure 3:**
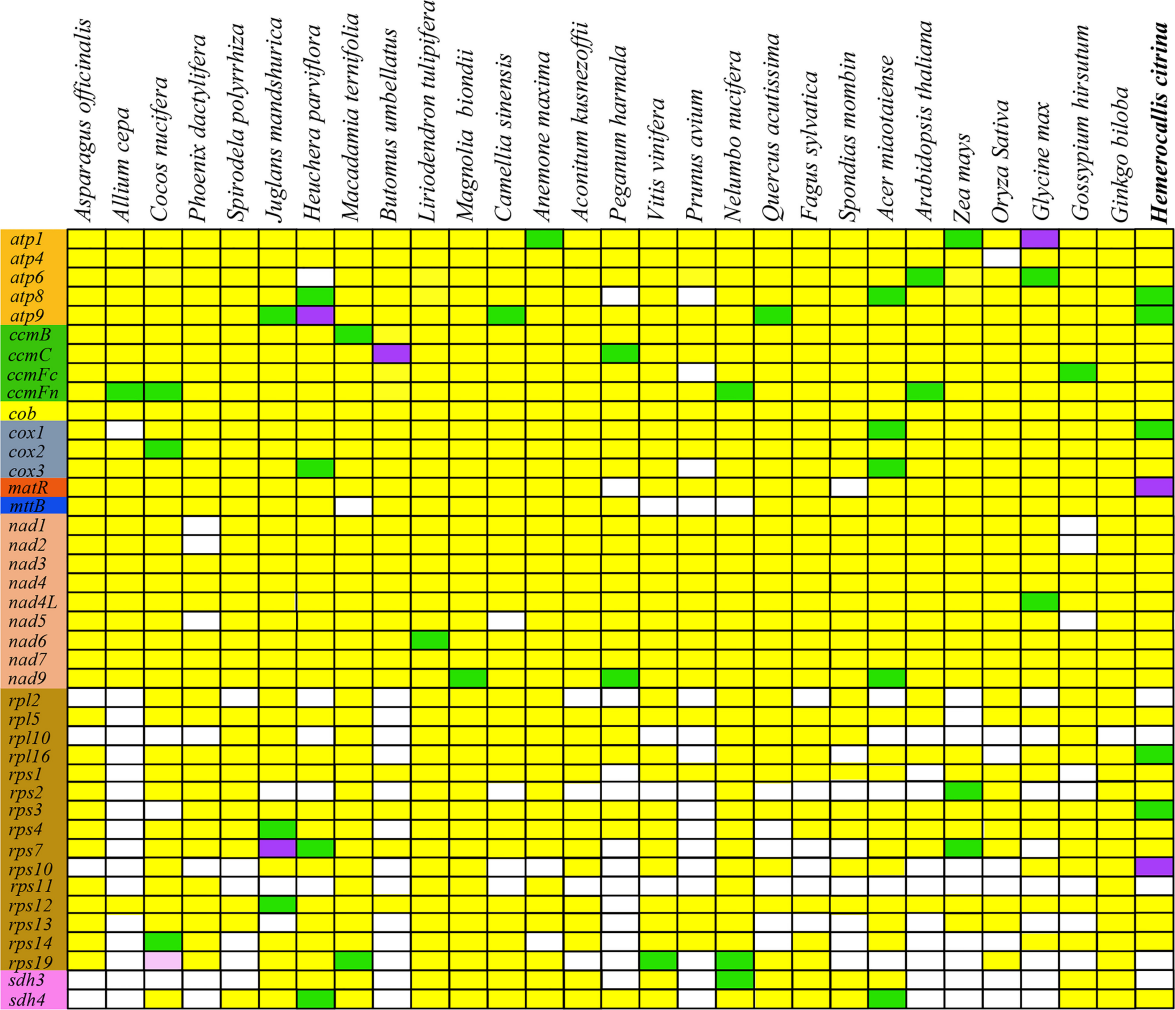
Distribution of PCGs in plant mitogenomes. White boxes indicate that the gene is absent in the mitogenome. The remaining colors of boxes indicate that one (yellow), two (green), three (purple), or five (pink) copies exist in the genomes.

The mitogenome gene content and order vary during the evolution of higher plants. Frequent occurrence of PCG loss that is extensively found in the course of evolution creates diversity in plant mitogenomes. To investigate gene loss in the *H. citrina* mitogenome, we compared its gene content with that of the other 29 species. As exhibited in **Figure 3**, the genes in the categories of Complex I, Complex III, Complex V, cytochrome c biogenesis, and maturases are conserved among these mitogenomes. The conserved genes relevant to mt respiration and energy synthesis, excluding the *sdh3* and *sdh4* encode subunits of mt Complex II, are essential to mt function. Consequently, variation of ribosomal protein genes appears to be the dominant factor in the high diversity of the gene content among plant mitogenomes.

In order to investigate the gene organization in *H. citrina*, the selected 28 species were used for mitogenome comparison and calculation of syntenic gene clusters. A total of six conserved gene clusters (i.e., *rps3-rpl16*, *atp4-nad4L*, *rrn18-rrn5*, *rps12-nad3*, *cob-rpl5*, and *atp1-atp9*) were identified in the *H. citrina* mitogenomes (**Supplementary Figure 1**, **Supplementary Table 3**). Furthermore, the cluster *cox3-sdh4* was primarily present in dicotyledons, but rarely occurred in monocotyledons. Interestingly, the conserved cluster *nad1-matR* existed extensively in most species except *H. citrina.* It is remarkable, however, that *H. citrina* was one of the few species containing the *atp1-atp9* gene cluster, similar to the other three monocotyledons (i.e., *A. officinalis*, *P. dactylifera*, and *Butomus umbellatus*). Among the dicotyledons, only *Aconitum kusnezoffii* had the cluster *atp1-atp9.*

### Identification of repeat sequences

Repeat sequences are characterized as a high degree of polymorphism and extensive presence in plant mitogenomes, which are important factors for genomic structural variances. The specific forms of repeat sequences are characterized primarily as simple sequence repeats (SSRs), tandem repeats, and dispersed repeats. For the investigation of *H. citrina*, we identified 530 repeat sequences with 379, 24, and 127 distributed among SSRs, tandem repeats, and dispersed repeats, respectively. The SSRs in the *H. citrina* mitogenome were classified as 40.6% monomers, 37.2% dimers, 6.9% trimers, 14.0% tetramers, 1.0% pentamers, and 0.3% hexamers (**Supplementary Table 4**). The mononucleotide repeats of A/T were determined to be the most extensive repeat type with 133 repeats identified, followed by the dinucleotide repeats of AG/CT with 83 repeats. Polynucleotide repeats accounted for a small proportion and were only found in the intronic or intergenic regions, apart from a single tetranucleotide repeat of AATG/ATTC identified in the coding region (*rps3*). As illustrated in **Supplementary Table 5**, the 24 tandem repeats observed in the *H. citrina* mitogenome varied between 25 and 86 bp in length, and most of them contained two copies of the sequence. These tandem repeats, without exception, were localized in the intergenic spacers.

In addition to the above two types of repeat, dispersed repeats (repeat unit >30) were analyzed further, resulting in 35,337 bp occupying 7.5% of the genome. The 127 dispersed repeats that were identified consisted of 59 forward repeats and 68 palindromic repeats, and a majority of them were 30~49 bp in length (**Supplementary Table 6**). There were nine such repeats greater than 100 bp, of which only three were greater than 1 kb (**Supplementary Table 7**). Simultaneously, we compared *H. citrina* with the other five monocotyledons to further explore the position of repeats in the mitogenome. The results suggested that the number of repeats counted in the six comparison species ranged from 38 in *Spirodela polyrrhiza* to 232 in *Zea mays* (**Supplementary Figure 2**, **Supplementary Table 8**). Interestingly, the total length of the repeats generally correlates with their mitogenome size. *C. nucifera* has the largest mitogenome (678,653 bp) among these species, and its total repeats cover 81,999 bp, occupying 12.1% of the total size of the mitogenome. In contrast, the mitogenome of *S. polyrrhiza* is only one-third the size of that of *C. nucifera*, and its repeats are the minimum among the comparative species, at 3,129 bp long and making up 1.4% of the genome size. Analyses of large repeats (>1 kb) implied that species with large mitogenomes were more abundant in large repeats. Eleven large repeats covering 68,972 bp were detected in *C. nucifera*, followed by the four large repeats of *Z. mays* (48,168 bp). However, no large repeats were found in the *S. polyrrhiza* mitogenome. The moderately sized *H. citrina* genome has repeats with a longer length than those found in the similar genome size of *A. officinalis*, mainly resulting from the difference in the number of large repeats.

### Determination of the codon usage pattern

In *H. citrina*, the majority of PCGs use NTG as their start codon, in which 28 unique genes have the standard ATG start codon, whereas the other two genes harbor the initiation codons GTG for *rpl16* and CTG for *sdh4.* Other than that, the remaining three genes (*nad1*, *nad4L*, and *rps10*) initiate with ACG (**Supplementary Table 9**). The complete PCGs in this mitogenome consist of three termination codon types, of which 17, 12, and 7 unique genes utilize the termination codons TAA, TGA, and TAG, respectively. The codon usage behavior revealed that Leucine (Leu) and Serine (Ser) were the most frequently utilized amino acids in *H. citrina*, whereas, as in other plant mitogenomes, Tryptophane (Trp) and Cysteine (Cys) were the least popular residues (**Supplementary Figure 3**). Furthermore, the vast majority of amino acid residues were highly conserved in dicotyledonous (*N. nucifera* and *Vitis vinifera*) and monocotyledonous (*H. citrina*, *A. officinalis*, *A. cepa*, *C. nucifera*, *S. polyrrhiza*, and *Z. mays*) plants based on interspecies alignment. Interestingly, codon usage showed a significant bias towards five amino acid residues (i.e., Phenylalanine (Phe), Leu, Proline (Pro), Ser, and Trp) in angiosperms and gymnosperms (*Cycas taitungensis* and *G. biloba*). The investigation of relative synonymous codon usage (RSCU) indicated that all possible codons were exhibited in the *H. citrina* PCGs (**Supplementary Figure 4**). Excluding the stop codons, the *H. citrina* mitogenome contains a total of 13,549 codons. The most preferentially used codons in this genome are A-ended or U-ended codons, which have RSCU values greater than 1, with the exception of Ser (AGU). This AT bias might influence codon usage in proteins, a phenomenon found in previously researched mitogenomes.

### Selection pressure analysis

**Figure 4:**
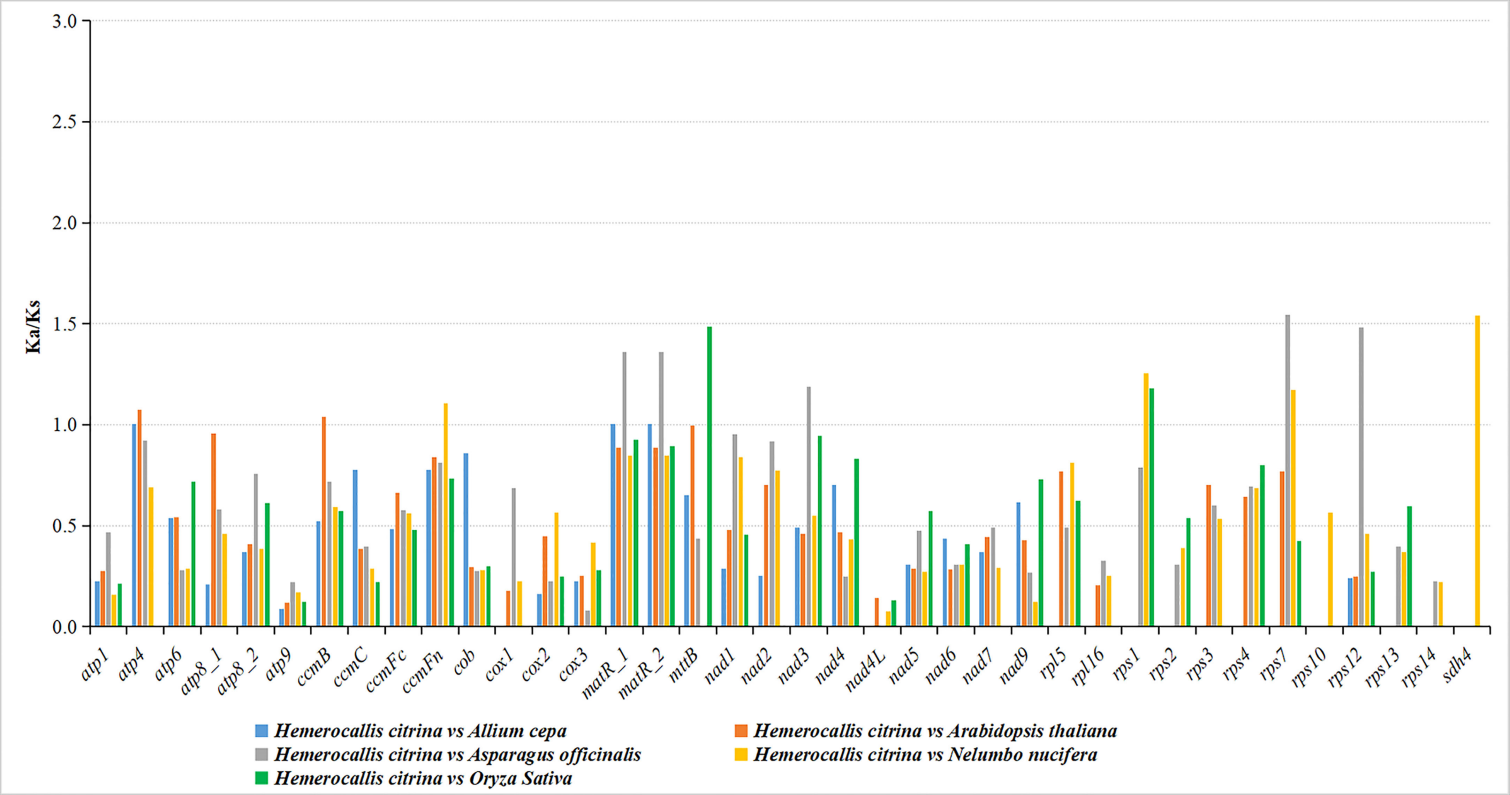
Comparison of the Ka/Ks ratios for PCGs in *H. citrina* and four other species.

The ratio of nonsynonymous substitutions (Ka) to synonymous substitutions (Ks) is a suitable test of the neutral evolutionary model to infer the magnitude and direction of natural selection working on homologous PCGs among diverse species. In our research, the Ka/Ks values were inspected for all 36 unique PCGs after contrasting the *H. citrina* mitogenome with those of *A. cepa*, *A. officinalis*, *A. thaliana*, *N. nucifera*, and *O. sativa.* As shown in **Figure 4**, most of the Ka/Ks ratios were less than 1, implying that the majority of PCGs had undergone purifying selection during the evolutionary process. Nearly all of the Complex I-V genes showed stabilizing selection, suggesting that these genes exhibited high conservation in *H. citrina* and the selected species during evolution. Three genes (*atp4*, *nad3*, and *sdh4*) encoding Complex subunit proteins were subject to positive selection with Ka/Ks values >1. Additionally, high Ka/Ks ratios were determined in some other mt genes, involving *ccmB*, *ccmFn*, *matR*, *mttB*, *rps1*, *rps7*, and *rps12*. As many as four positive selection genes were observed in the comparison of *H. citrina* with *A. officinalis* and *N. nucifera*, suggesting that those mt genes may have suffered from diverse selection pressures after diverging from their last common ancestor. Interestingly, none of the PCGs had Ka/Ks values greater than 1, which only existed between *H. citrina* and *A. cepa.*

### Prediction of RNA editing sites

**Figure 5:**
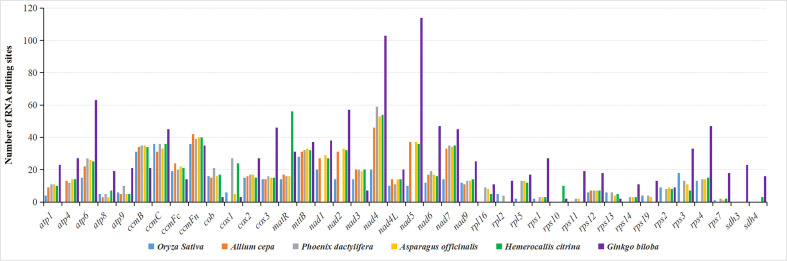
Prediction of RNA editing sites in the *H. citrina* mitogenome and five other species.

For the identification of *H. citrina*, all 45 PCGs were subject to RNA editing, and a total of 679 sites were predicted. Among the predicted editing events, the conversions carried out were uniformly post-transcriptional cytidine (C) to uridine (U) editing. This identified 679 sites where the RNA editing bases were substituted at the first 2-base positions of the codon. The frequency of the editing events in PCGs varied greatly, ranging from two editing sites in *rps7* to 56 editing sites in *matR.* As shown in **Figure 5**, RNA editing was also compared across six species and ranged from 491 sites in *O. sativa* to 1,405 sites in *G. biloba.* The RNA editing site was relatively conserved among angiospermous mitogenomes, and that of *H. citrina* showed high similarity with that of *A. officinalis*, indicating that they share extremely conserved PCGs. The occurrence of RNA editing is obviously influenced by gene type, and the cytochrome c biogenesis and Complex I genes are more prone to RNA editing. Furthermore, there were ten cases involving the genes *ccmB*, *ccmC*, *ccmFc*, *ccmFn*, *cox2*, *cox3*, *mttB*, *nad4L*, *nad9*, and *rps12*, in which the number of edits was highly conserved in the provided angiosperms. Notably, the number of predicted sites of almost all genes in the gymnosperm (*G. biloba*) was markedly higher than that in angiosperms. However, *matR* was one of the exceptions, and the highest frequency of RNA editing within this gene renders *H. citrina* stand out in comparison to the other species.

### Phylogenetic analysis

**Figure 6:**
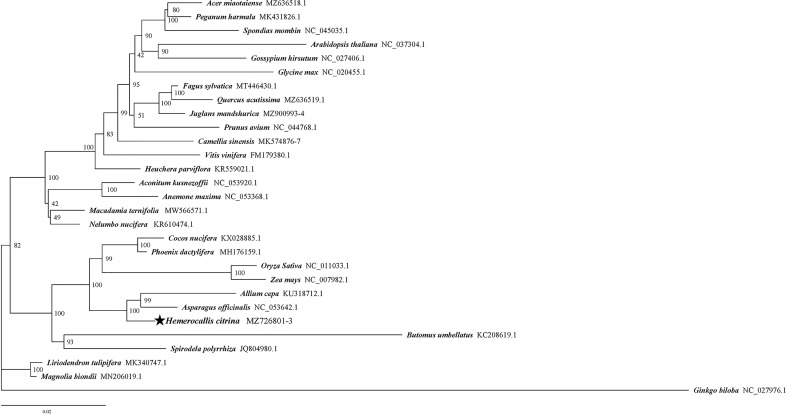
Maximum likelihood phylogenetic tree for *H. citrina* according to 29 complete mitogenomic sequences, with *Ginkgo biloba* as an outgroup. The bootstrap index value in which the associated taxa clustered together is indicated next to the branches, and the NCBI accession numbers are listed beside the abbreviations of the species.

A phylogenetic analysis of 29 complete mitogenomes was proceeded to explore the phylogenetic position of *H. citrina.* The results suggested that the majority of nodes had high bootstrap values for the maximum likelihood (ML) tree. Importantly, *H. citrina* is evolutionarily close to the species *A. cepa* (Amaryllidaceae) and *A. officinalis* (Asparagaceae) based on 100% bootstrap values (**Figure 6**). Our phylogenetic analysis also strongly indicated the divergence of dicotyledons and monocotyledons, as well as the divergence of angiosperms and gymnosperms.

## Discussion

Since the initial endosymbiosis event, mitogenomes have undergone significant and divergent changes in genome structure (Smith and Keeling, 2015). With the worldwide sequencing and assembly of mitogenomes in land plants, the true physical structure of the mitogenome is gradually emerging in multifarious forms, such as single circular molecule, multiple circular, linear, branched or composite structures (Unseld et al., 1997; Notsu et al., 2002; Alverson et al., 2011; Sloan et al., 2012; Jackman et al., 2020; Li et al., 2021a). To explore these challenging mitogenome structures in our study, we adopted a mixed assembly strategy, integrating Illumina short-read and Oxford Nanopore long-read sequencing to complete the mitogenome of *H. citrina.* The genome assembly presents a multiple chromosomal structure, composed of three distinct circular molecules with lengths of 45,607 bp, 239,991 bp, and 182,864 bp (**Figures 1B-D**). The newly sequenced *H. citrina* mitogenome is close to the average genome size (394.9 kb) and GC content (43.95%) of published land plants (Wu et al., 2020). Similarly, the genomes of *Sitka spruce* (Jackman et al., 2020), *Leucaena trichandra* (Kovar et al., 2018), and *Lactuca sativa* (Kozik et al., 2019) were successfully sequenced and precisely assembled using the combined approach. The resulting research further confirmed that second- and third-generation sequencing combined assemblies promise to obtain better and more reliable *de novo* assembly results for plant mitogenomes.

Plant mitogenomes are fascinating attributing to their highly conserved gene content and sequences, which are noted as having the slowest evolutionary rate of the three sets of plant genomes (nuclear, cp, and mt genomes). Despite differences in the number of mt genes across different plants, the genes required to maintain the major mt functions of respiration and energy synthesis are highly conserved. In general, the Complex genes responsible for mt respiration and metabolism are quite stable, with the exception of the succinate dehydrogenase (*sdh*) genes that encode subunits of mt Complex II. In comparing the gene content of different species, we found that *sdh3* was lost in monocotyledons during evolution (**Figure 3**). In addition, *sdh4* was also scarcer in the mitogenomes of most monocot plants. Our findings revealed that only *sdh4* was present in the *H. citrina* mitogenome, which is consistent with results previously reported for two other monocot plants, *C. nucifera* (Aljohi et al., 2016) and *S. polyrrhiza* (GenBank accession: JQ804980.1). In contrast, the absence of *sdh* genes was observed only in a few dicotyledons. Consequently, the frequent loss of *sdh3* and *sdh4* tends to occur in monocotyledons. Furthermore, the plant mitogenome also harbors many ribosomal protein genes, which present substantial variation in sequence conservation and gene content across species (Liao et al., 2018). In our research of *H. citrina*, we annotated up to 11 unique ribosomal protein genes excluding the four genes *rpl2*, *rpl10*, *rps11*, and *rps19*. Surprisingly, in the mitogenome of *A. cepa* (Kim et al., 2016), there was only one ribosomal protein gene (*rps12*), which is far fewer than in most spermatophytes. The difference in the PCG content between *H. citrina* and *A. cepa* appears to be driven by the high diversity of ribosomal protein genes.

Although mitogenomic structures and sizes are highly fluid among spermatophytes, the generation of similar gene clusters by multiple recombination events often leads to conserved gene organizations across large phylogenetic scales (Overbeek et al., 1999; Gui et al., 2016). In the comparison of gene clusters in *H. citrina* and 28 other plants, six syntenic gene clusters were explored in *H. citrina* (**Supplementary Figure 1**, **Supplementary Table 3**). Five of those conserved clusters (*rps3-rpl16*, *atp4-nad4L*, *rrn18-rrn5*, *rps12-nad3*, and *cob-rpl5*) were identified in almost all the compared species and might date back to their original mitogenomes. The *cox3-sdh4* gene cluster seemed to show a preference for distribution in dicotyledons, indicating its existence in the angiosperm ancestor followed by rapid degeneration due to substantial genome rearrangements. Notably, the cluster *nad1-matR* emerged extensively in most species but was absent from *H. citrina*, suggesting that recombination events occurred frequently during the evolution of the *H. citrina* mitogenome. Generally speaking, gene orders are more similar in species with close evolutionary relationships. The sharing of the rare *atp1-atp9* cluster by *H. citrina* and three other monocotyledons (*A. officinalis*, *P. dactylifera*, and *B. umbellatus*), appears to support their close relationship during the evolution of the plant mitogenomes.

Plant mitogenomes contain sizeable fractions of repeat sequences that are assumed to be a vital factor in genome structure and size variations. Previous researches have documented that repeats in mt present high levels of polymorphism and serve as sites of intra- or intergenomic recombination to produce multiple alternative arrangements (Fujii et al., 2010; Sloan, 2013; Gualberto et al., 2014). In *H. citrina*, the repeat sequences had a size of 39,294 kb and accounted for 8.4% of the mitogenome. However, more than 76% of the repeats were primarily comprised of SSRs and tandem repeats and only occupied 0.9% of the genome (**Supplementary Tables 4, 5**). In contrast, the 127 dispersed repeats identified amounted to 35,337 kb and occupied 7.5% of the *H. citrina* mitogenome (**Supplementary Table 8**). From the comparison of *H. citrina* with other monocots (**Supplementary Figure 2**, **Supplementary Table 8**), we were encouraged to acquire evidence that the scale of dispersed repeats largely correlates with the size of their mitogenome despite their poor conservation across species. Although only three large repeats (>1 kb) were detected in *H. citrina*, the presence of large segmental duplications possibly led to mitogenome expansion. In conclusion, the abundant repetitive sequences may accelerate the rearrangement of the *H. citrina* mitogenome.

As a post-transcriptional processing event, RNA editing is pervasive among higher plants, contributing to maintaining the amino acid sequence conservation of important functional proteins in mt. The occurrence of RNA editing generally converts cytidine (C) to uridine (U), sometimes causing the alteration of initiation and termination codons, but more often creating internal codons with powerful functional correlations that alter coding messages (Handa, 2003). For the *H. citrina* investigation, 679 RNA editing sites within all 45 PCGs were inferred throughout the mitogenome. Unsurprisingly, 100% of the potential editing sites were the expected C-to-U type, which also correspond to certain activities in most spermatophytes. The majority of editing sites led to the alteration of internal codons, and in particular the amino acids exhibited a significant preference in 43.0% (292 sites) of the edits and were transformed to Leucine after RNA editing. This particular phenomenon has also been documented in a previous report (Cheng et al., 2021). Serine and Proline were more susceptible to editing in *H. citrina*, occupying 37.6% (255 sites) and 34.0% (231 sites), respectively, of the identified edits, which is consistent with the conclusions of a previous study (Sloan and Taylor, 2010). Furthermore, the regulation process created initiation codons by altering ACG to AUG for the transcripts of *nad1*, *nad4L*, and *rps10.* In a similar pattern, *atp6* and *ccmFc* used alternative termination codons by editing CAA to UAA or CGA to UGA. The prediction of RNA editing events could provide possibilities for deciphering gene functions with novel codons. In closely related lineages, RNA editing sites are universally thought to be conserved, such as in Cruciferae (Handa, 2003) and Cucurbitaceae (Alverson et al., 2010). In a comparison of multiple species, the type and frequency of RNA editing in *H. citrina* were very similar to those of *A. officinalis* (**Figure 5**), which provided further evidence of their close evolutionary relationship.

When discussing the evolutionary dynamics of PCG sequences for genetically close species, it is essential to analyze the nucleotide substitution rates to comprehensively understand the evolution of plant mt genes. The Ka/Ks ratio is typically applied to estimate the selective pressure to which specific mitogenome PCGs are subjected during evolution (Zhang et al., 2006). In general, most mt genes have undergone negative or neutral selection considering their high level of conservation, resulting in Ka/Ks values that do not exceed 1 (Bi et al., 2020; Cheng et al., 2021; Yu et al., 2022). From Ka/Ks analysis of all 36 unique PCGs from the *H. citrina* mitogenome with five other species, we detected that the majority of PCGs had undergone purifying selection during the evolutionary process (**Figure 4**). However, the Ka/Ks values of *atp4*, *nad3*, and *sdh4* were greater than 1, implying positive selections of these genes in *H. citrina*. Since the three genes encode the vital components of the mt Complex subunit, we conjecture that the adaptive evolution of *H. citrina* has been to improve respiratory electron transport and oxidative phosphorylation. An additional seven genes (*ccmB*, *ccmFn*, *matR*, *mttB*, *rps1*, *rps7*, and *rps12*) were identified as having been subjected to positive selection pressure in *H. citrina.* Genes *ccmFn* and *ccmB* are believed to participate in the cytochrome c maturation process of mt by affecting the export and metabolism of heme (Giegé et al., 2008; Verrier et al., 2008). The *matR* gene exists within *nad1* intron 4 as the only maturase retained in the angiosperm mt (Sultan et al., 2016), but the biological function of *matR* remains elusive. The product of *mttB* is a membrane transport protein that participates in proton transmembrane transfer activity (Gaudet et al., 2011). Additionally, genes *rps1*, *rps7*, and *rps12* encode the small mitoribosomal subunit proteins in various biological pathways. Altogether, these genes that have undergone positive selection might have developed novel functions and played a crucial role in the evolution of *H. citrina*. Interestingly, the Ka/Ks values observed for *atp9* were notably lower than those for all other PCGs in the *H. citrina* investigation, implying that strong negative selection was acting on *atp9* to maintain its indispensable function in these mitogenomes. The *atp9* gene is considered to be involved in proton transport (Gaudet et al., 2011), and, more importantly, it is known to be intimately associated with cytoplasmic male sterility (Reddemann and Horn, 2018). We were surprised to find that the Ka/Ks values did not exceed 1 for all PCGs between *H. citrina* and *A. cepa*, which hints strongly at their close evolutionary relationship.

## Conclusions

Currently, we assembled and depicted the complete mitogenome of *H. citrina* based on a strategy using Oxford Nanopore long-read and Illumina short-read sequencing. The multiple chromosomal structure of *H. citrina* broadens our understanding of structural complexity in plant mitogenomes. Extensive analyses were performed by comparing the genomic characteristics of the *H. citrina* mt with those of multiple plant mitogenomes, as illustrated by the conservation of the gene content, syntenic gene clusters, repeat sequences, codon usage preferences, selection pressure, prediction of RNA edits, and phylogenetic tree construction. The enormous genomic variability discovered sheds new light on the evolutionary process of higher plant mitogenomes. In summary, the exhaustive nature of the *H. citrina* mitogenome should offer deeper insights into the evolution of the genus *Hemerocallis* and should serve as a fundamental reference for further molecular breeding.

## Data availability statement

The datasets presented in this study can be found in online repositories. The names of the repository/repositories and accession number(s) can be found below: https://www.ncbi.nlm.nih.gov/nuccore/MZ726801.1/, MZ726801.1, https://www.ncbi.nlm.nih.gov/nuccore/MZ726802.1/, MZ726802.1, https://www.ncbi.nlm.nih.gov/nuccore/MZ726803.1/, MZ726803.1, https://www.ncbi.nlm.nih.gov/bioproject/PRJNA752889, PRJNA752889, https://www.ncbi.nlm.nih.gov/sra/SRR15370204, SRR15370204, https://www.ncbi.nlm.nih.gov/sra/SRR15370205, SRR15370205, https://www.ncbi.nlm.nih.gov/biosample/SAMN20667040/, SAMN20667040.

## Author contributions

KZ and YW conceived the idea. KZ and XS performed data analysis and wrote the manuscript. YW, XZ, and ZH supervised the research and revised the manuscript. All authors contributed to the article and approved the submitted version.

## Funding

This research was funded by Shanxi Province Science Foundation for Youths (Grant No. 201901D211426), Datong Science and Technology Bureau Applied Basic Research Project (Grant No. 2020137), Youth Science and Technology Innovation Project of Tianjin Academy of Agricultural Sciences (Grant No. 2022014).

## Acknowledgments

The authors thank Shenzhen Huitong Biotechnology Co., Ltd., China for sequencing and MDPI for English language editing.

## Conflict of interest

The authors declare that the research was conducted in the absence of any commercial or financial relationships that could be construed as a potential conflict of interest.

## Publisher’s note

All claims expressed in this article are solely those of the authors and do not necessarily represent those of their affiliated organizations, or those of the publisher, the editors and the reviewers. Any product that may be evaluated in this article, or claim that may be made by its manufacturer, is not guaranteed or endorsed by the publisher.
