# Supplementary material for: Deciphering the mitochondrial genome of Hemerocallis citrina (Asphodelaceae) using a combined assembly and comparative genomic strategy
Source: Front Plant Sci. 2022 Nov 18;13:1051221. doi: 10.3389/fpls.2022.1051221 (PMC9715983; doi:10.3389/fpls.2022.1051221)
Supplement: Supplementary file 1 [file DataSheet_1.docx]

Supplementary Material

## Supplementary Tables

**TABLE 1.** The abbreviations and NCBI accession numbers of studied mitogenomes.

| **Species** | **Abbreviations** | **NCBI Accession Numbers** |
| --- | --- | --- |
| *Acer miaotaiense* P.C.Tsoong | *A. miaotaiense* | MZ636518.1 |
| *Aconitum kusnezoffii* Rchb. | *A. kusnezoffii* | MW013323.1 |
| *Allium cepa* L. | *A. cepa* | KU318712.1 |
| *Anemone maxima* Nakai | *A. maxima* | MT568500.1 |
| *Arabidopsis thaliana* (L.) Heynh | *A. thaliana* | BK010421.1 |
| *Asparagus officinalis* L. | *A. officinalis* | MT483944.1 |
| *Butomus umbellatus* L. | *B. umbellatus* | KC208619.1 |
| *Camellia sinensis* (L.) Kuntze | *C. sinensis* | MK574876.1, MK574877.1 |
| *Cocos nucifera* L. | *C. nucifera* | KX028885.1 |
| *Fagus sylvatica* L. | *F. sylvatica* | MT446430.1 |
| *Ginkgo biloba* L. | *G. biloba* | KM672373.1 |
| *Glycine max* (L.) Merr. | *Glycine max* | NC_020455.1 |
| *Gossypium hirsutum* L. | *G. hirsutum* | NC_027406.1 |
| *Hemerocallis citrina* Baroni | *H. citrina* | MZ726801.1, MZ726802.1, MZ726803.1 |
| *Heuchera parviflora* Bartl. | *H. parviflora* | KR559021.1 |
| *Juglans mandshurica* Maxim. | *J. mandshurica* | MZ900993.1, MZ900994.1 |
| *Liriodendron tulipifera* L. | *L. tulipifera* | MK340747.1 |
| *Macadamia ternifolia* F.Muell | *M. ternifolia* | MW566571.1 |
| *Magnolia biondii* Pamp. | *M. biondii* | MN206019.1 |
| *Nelumbo nucifera* Gaertn. | *N. nucifera* | KR610474.1 |
| *Oryza sativa* L. | *O. sativa* | NC_011033.1 |
| *Peganum harmala* L. | *P. harmala* | MK431826.1 |
| *Phoenix dactylifera* L. | *P. dactylifera* | MH176159.1 |
| *Prunus avium* (L.) L. | *P. avium* | MK816392.2 |
| *Quercus acutissima* Carruth. | *Q. acutissima* | MZ636519.1 |
| *Spirodela polyrrhiza* (L.) Schleid. | *S. polyrrhiza* | JQ804980.1 |
| *Spondias mombin* L. | *S. mombin* | MN057920.1 |
| *Vitis vinifera* L. | *V. vinifera* | FM179380.1 |
| *Zea mays* L. | *Z. mays* | NC_007982.1 |

**TABLE 2.** Composition of the *H. citrina* mitogenome.

| Category | Feature | Number | Length (bp)  Proportion in Genome (%) | Number | Length (bp)  Proportion in Genome (%) | Number | Length (bp)  Proportion in Genome (%) |
| --- | --- | --- | --- | --- | --- | --- | --- |
|  |  | Molecule 1 | | Molecule 2 | | Molecule 3 | |
| Genome | G+C |  | 20,249 (44.40%) |  | 108,140 (45.06%) |  | 83,532 (45.68%) |
| Genes |  | | |  |  |  |  |
|  | Protein coding | 5 | 4,683 (10.27%) | 27 | 21,604 (9.00%) | 16 | 14,495 (7.93%) |
|  | rRNA |  |  |  |  | 4 | 7,300 (3.99%) |
|  | mt-derived tRNA |  |  | 4 | 299 (0.12%) | 9 | 689 (0.38%) |
|  | cp-derived tRNA |  |  | 1 | 73 (0.03%) | 3 | 221 (0.12%) |
| Introns |  | | |  |  |  |  |
|  | *cis*-spliced |  |  | 10 | 11,094 (4.62%) | 7 | 12,046 (6.59%) |
|  | *trans-*spliced |  |  | 5 | not know | 2 | not know |
| Repeats |  | | |  |  |  |  |
|  | Larger repeats (> 1000) |  |  | 2 | 13,005 (5.42%) | 1 | 16,460 (9.00%) |
|  | Medium repeats (> 100 , < 999) | 1 | 176 (0.39%) | 1 | 112 (0.05%) | 4 | 489 (0.27%) |
|  | Short repeats (> 20, < 99) | 1 | 36 (0.08%) | 39 | 1549 (0.65%) | 78 | 3,510 (1.92%) |

**TABLE 3.** Distribution of gene clusters in *H. citrina* and other higher plant mitogenomes.

| **Species** | ***(rps19)-rps3-rpl16*** | ***atp4-nad4L*** | ***rrn18-rrn5*** | ***rps12-nad3*** | ***cob-(rps14)-rpl5*** | ***rps13-nad1*** | ***(nad5)-nad1-matR*** | ***(atp8)-cox3-sdh4*** | ***nad6-rps4*** | ***rps10-cox1*** | ***atp1-atp9*** |
| --- | --- | --- | --- | --- | --- | --- | --- | --- | --- | --- | --- |
| *Asparagus officinalis* L. | + | + | + | + | + | + | + | / | - | / | + |
| *Allium cepa* L. | / | + | - | + | / | / | + | / | / | / | - |
| *Cocos nucifera* L. | / | + | + | + | + | + | + | + | - | - | - |
| *Phoenix dactylifera* L. | + | + | + | + | + | / | / | / | - | / | + |
| *Spirodela polyrrhiza* (L.) Schleid. | + | + | + | + | + | + | + | + | - | / | - |
| *Juglans mandshurica* Maxim. | + | + | + | + | + | / | + | + | + | - | - |
| *Heuchera parviflora* Bartl. | + | + | + | + | + | + | + | + | + | + | - |
| *Macadamia ternifolia* F.Muell | + | + | + | + | + | + | + | + | - | + | - |
| *Butomus umbellatus* L. | / | + | + | - | / | / | + | + | / | / | + |
| *Liriodendron tulipifera* L. | + | + | + | + | + | - | + | + | - | - | - |
| *Magnolia biondii* Pamp. | + | + | - | + | + | + | + | + | - | - | - |
| *Camellia sinensis* (L.) Kuntze | + | + | + | + | + | + | + | + | + | - | - |
| *Anemone maxima* Nakai | + | + | + | - | + | + | + | + | - | / | - |
| *Aconitum kusnezoffii* Rchb. | + | + | + | + | + | + | + | + | - | + | + |
| *Peganum harmala* L. | + | + | - | / | + | / | / | + | - | / | - |
| *Vitis vinifera* L. | + | + | + | + | + | - | + | + | + | - | - |
| *Prunus avium* (L.) L. | / | + | + | + | + | + | + | / | / | / | - |
| *Nelumbo nucifera* Gaertn. | + | + | + | + | + | + | + | + | - | + | - |
| *Quercus acutissima* Carruth. | + | + | + | + | + | / | + | + | / | + | - |
| *Fagus sylvatica* L. | + | + | + | + | + | / | + | + | + | / | - |
| *Spondias mombin* L. | + | + | + | + | + | / | / | + | + | + | - |
| *Acer miaotaiense* P.C.Tsoong | + | + | + | + | + | + | + | + | + | - | - |
| *Arabidopsis thaliana* (L.) Heynh | + | + | + | + | + | / | + | + | - | / | - |
| *Zea mays* L. | + | - | + | + | / | + | + | / | - | / | - |
| *Oryza sativa* L. | + | / | + | + | + | + | + | / | - | / | - |
| *Glycine max* (L.) Merr. | + | - | + | + | + | / | + | / | - | + | - |
| *Gossypium hirsutum* L. | + | - | + | + | + | / | / | + | - | + | - |
| *Ginkgo biloba* L. | + | - | + | + | + | - | + | + | - | + | - |
| *Hemerocallis citrina* Baroni | + | + | + | + | + | - | - | - | - | - | + |

**+**, indicates that the cluster exists in plant mtDNA; **-**, indicates that the cluster is absent in plant mtDNA; **/**, indicates that the cluster is lost.

**TABLE 4.** Distribution of SSRs in the *H. citrina* mitogenome.

| **SSRs Motif Type** | **Number of SSRs** | | | **Total** | **Proportion (%)** |
| --- | --- | --- | --- | --- | --- |
|  | Molecule 1 | Molecule 2 | Molecule 3 |  |  |
| Monomer | 15 | 77 | 62 | 154 | 40.6 |
| Dimer | 17 | 76 | 48 | 141 | 37.2 |
| Trimer | 1 | 17 | 8 | 26 | 6.9 |
| Tetramer | 6 | 30 | 17 | 53 | 14.0 |
| Pentamer | - | 3 | 1 | 4 | 1.0 |
| Hexamer | - | 1 | - | 1 | 0.3 |
| Total | 39 | 204 | 136 | 379 | 100 |

-, absent.

**TABLE 5.** List of tandem repeats in the *H. citrina* mitogenome.

| **Genome** | **No.** | **Size (bp)** | **Start** | **End** | **Repeat × Copy Number** | **Location** |
| --- | --- | --- | --- | --- | --- | --- |
| Molecule 1 | 1 | 38 | 230 | 267 | (CTTTCGAACTTAACTTATA) × 2 | IGS (*atp8*, *atp9*) |
|  | 2 | 31 | 4898 | 4928 | (TGTCTTTAAGTATTT) × 2.1 | IGS (*atp1*, *atp8*) |
|  | 3 | 30 | 44292 | 44321 | (TTGACCAGCCAACCG) × 2 | IGS *(atp8*, *atp9)* |
| Molecule 2 | 4 | 27 | 22772 | 22798 | (TAGCTACTACTAC) × 2.1 | IGS (*atp4*, *nad2*-exon1) |
|  | 5 | 27 | 52286 | 52312 | (AGTAGTAGTAGCT) × 2.1 | IGS (*rps12*, *ccmC*) |
|  | 6 | 25 | 56990 | 57014 | (GCTACTACTACTA) × 1.9 | IGS (*ccmC*, *trnK*) |
|  | 7 | 25 | 61662 | 61686 | (ATTTGAACTAGA) × 2.1 | IGS (*trnK*, *trnC*) |
|  | 8 | 26 | 64054 | 64079 | (TAGTAGTAGTAGC) × 2 | IGS (*trnK, trnC*) |
|  | 9 | 36 | 67127 | 67162 | (TTCCTATAACCAAACCTAA) × 1.9 | IGS (*trnC*, *nad1*-exon1) |
|  | 10 | 79 | 92628 | 92706 | (AATATCATGATCGGGTCGACCAGGCCAGATCATAAGTGA) × 2 | IGS (*trnC*, *nad1*-exon1) |
|  | 11 | 44 | 106619 | 106662 | (TGAAGGCTA) × 4.9 | IGS (*nad1*-exon1, *rps1*) |
|  | 12 | 43 | 151023 | 151065 | (AAGGCTAAGAAAAAGAAACCG) × 2 | IGS (*rpl16*, *trnE*) |
|  | 13 | 64 | 154873 | 154936 | (GCTTACCTTAGCCCAACCATACGTCCTACT) × 2.1 | IGS (*rpl16*, *trnE*) |
|  | 14 | 40 | 161584 | 161623 | (ACTAAGTATATTAAGGGTC) × 2.1 | IGS (*rpl16*, *trnE*) |
|  | 15 | 25 | 205115 | 205139 | (TTCTCCTCC) × 2.8 | IGS (*nad1*-exon4, *cox2*-exon1) |
|  | 16 | 38 | 233131 | 233168 | (CTTTCGAACTTAACTTATA) × 2 | IGS (*rps2*, *atp9*) |
| Molecule 3 | 17 | 28 | 57855 | 57882 | (TTCCAGCGCTCTTG) × 2 | IGS (*nad*-exon5, *rns*) |
|  | 18 | 77 | 70057 | 70133 | (ACTATA) × 12.8 | IGS (*rps4*, *trnM*) |
|  | 19 | 86 | 70062 | 70147 | (ACTATAACTATAATATA) × 5.1 | IGS (*rps4*, *trnM*) |
|  | 20 | 30 | 74623 | 74652 | (TATATTCTATATA) × 2.3 | IGS (*trnS*, *trnI*) |
|  | 21 | 46 | 87689 | 87734 | (TAAGGATCATATGTGCTATGAGT) × 2 | IGS (*nad6*, *nad*-exon3) |
|  | 22 | 26 | 140645 | 140670 | (TAGTAGTAGTAGC) × 2 | IGS (*trnF*, *ccmFn*) |
|  | 23 | 25 | 164488 | 164512 | (AGTAGTAGTAGCT) × 1.9 | IGS (*trnH*, *atp6*) |
|  | 24 | 28 | 174996 | 175023 | (TTCCAGCGCTCTTG) × 2 | IGS (*atp6*, *rns*) |

IGS, intergenic pacers.

**TABLE 6.** Statistics of dispersed repeats in the *H. citrina* mitogenome.

| **Size (bp)** | **Number of Forward Repeats** | **Number of Palindromic Repeats** |
| --- | --- | --- |
| 30-49 | 41 | 51 |
| 50-69 | 9 | 6 |
| 70-99 | 3 | 8 |
| 100-149 | 3 | 1 |
| 150-199 | 1 | 1 |
| 200-999 | 0 | 0 |
| ≥1000 | 2 | 1 |

**TABLE 7.** Dispersed repeats (repeat unit > 30 bp) in the *H. citrina* mitogenome.

| **Number** | **Size (bp)** | **Start of Copy1** | **Start of Copy2** | **Type*** |
| --- | --- | --- | --- | --- |
| R1 | 16,460 | 49264 | 166405 | F |
| R2 | 8,794 | 195717 | 224253 | F |
| R3 | 4,211 | 138737 | 235773 | P |
| R4 | 176 | 5816 | 22582 | P |
| R5 | 161 | 96014 | 106287 | F |
| R6 | 113 | 92928 | 102083 | P |
| R7 | 112 | 62637 | 222447 | F |
| R8 | 110 | 36079 | 37696 | F |
| R9 | 105 | 28614 | 65934 | F |
| R10 | 84 | 140019 | 172800 | P |
| R11 | 84 | 172800 | 238618 | F |
| R12 | 76 | 24131 | 102080 | F |
| R13 | 75 | 65594 | 110821 | P |
| R14 | 75 | 110821 | 182735 | P |
| R15 | 73 | 24134 | 92968 | P |
| R16 | 72 | 28247 | 67962 | F |
| R17 | 72 | 65587 | 96097 | P |
| R18 | 72 | 65587 | 106370 | P |
| R19 | 72 | 96097 | 182728 | P |
| R20 | 72 | 106370 | 182728 | P |
| R21 | 65 | 16244 | 86010 | F |
| R22 | 65 | 59103 | 165177 | P |
| R23 | 65 | 96097 | 110831 | F |
| R24 | 65 | 106370 | 110831 | F |
| R25 | 65 | 165177 | 176244 | P |
| R26 | 65 | 22754 | 52266 | P |
| R27 | 64 | 45814 | 165179 | P |
| R28 | 63 | 45815 | 59103 | F |
| R29 | 63 | 45815 | 176244 | F |
| R30 | 63 | 97577 | 152902 | F |
| R31 | 56 | 58837 | 127648 | P |
| R32 | 56 | 127648 | 175978 | P |
| R33 | 53 | 97531 | 152855 | F |
| R34 | 50 | 24186 | 96120 | F |
| R35 | 50 | 24186 | 106393 | F |
| R36 | 49 | 24186 | 65587 | P |
| R37 | 49 | 24186 | 182728 | P |
| R38 | 49 | 85355 | 86005 | F |
| R39 | 46 | 26869 | 48790 | P |
| R40 | 46 | 40814 | 177579 | 46 |
| R41 | 45 | 5006 | 208687 | 45 |
| R42 | 45 | 28838 | 66149 | 45 |
| R43 | 44 | 16244 | 85360 | 44 |
| R44 | 44 | 86490 | 200804 | P |
| R45 | 44 | 86490 | 229340 | P |
| R46 | 44 | 138736 | 192608 | P |
| R47 | 43 | 22774 | 56990 | F |
| R48 | 43 | 52268 | 56990 | P |
| R49 | 43 | 192608 | 239941 | F |
| R50 | 43 | 16244 | 83749 | P |
| R51 | 43 | 83749 | 85360 | P |
| R52 | 43 | 83749 | 86010 | P |
| R53 | 43 | 129261 | 155297 | F |
| R54 | 42 | 8326 | 109509 | P |
| R55 | 42 | 24186 | 110854 | F |
| R56 | 42 | 83704 | 141496 | P |
| R57 | 42 | 85366 | 104579 | P |
| R58 | 42 | 85411 | 141495 | F |
| R59 | 41 | 83676 | 165150 | F |
| R60 | 41 | 83705 | 85412 | P |
| R61 | 41 | 19494 | 24454 | F |
| R62 | 40 | 86533 | 204402 | F |
| R63 | 40 | 86533 | 232938 | F |
| R64 | 40 | 64958 | 112434 | P |
| R65 | 40 | 112434 | 182099 | P |
| R66 | 39 | 88206 | 160473 | F |
| R67 | 39 | 23229 | 88209 | P |
| R68 | 39 | 59443 | 93658 | P |
| R69 | 39 | 62640 | 193804 | P |
| R70 | 39 | 193804 | 222450 | P |
| R71 | 38 | 56977 | 64055 | P |
| R72 | 38 | 9132 | 129265 | P |
| R73 | 38 | 9132 | 155301 | P |
| R74 | 38 | 16250 | 104583 | P |
| R75 | 38 | 86016 | 104583 | P |
| R76 | 37 | 83749 | 104584 | F |
| R77 | 37 | 70835 | 81393 | F |
| R78 | 36 | 86347 | 198950 | F |
| R79 | 36 | 86347 | 227486 | F |
| R80 | 36 | 14330 | 33424 | P |
| R81 | 36 | 85618 | 156913 | P |
| R82 | 36 | 108709 | 112600 | P |
| R83 | 36 | 109472 | 149918 | P |
| R84 | 35 | 11077 | 92933 | F |
| R85 | 35 | 11077 | 102156 | P |
| R86 | 34 | 50282 | 162907 | F |
| R87 | 34 | 162907 | 167423 | F |
| R88 | 34 | 86488 | 198987 | P |
| R89 | 34 | 86488 | 227523 | P |
| R90 | 33 | 96183 | 110622 | P |
| R91 | 32 | 91944 | 93678 | F |
| R92 | 32 | 119575 | 128182 | P |
| R93 | 32 | 198987 | 200816 | F |
| R94 | 32 | 198987 | 229352 | F |
| R95 | 32 | 200816 | 227523 | F |
| R96 | 32 | 227523 | 229352 | F |
| R97 | 32 | 15724 | 63231 | F |
| R98 | 32 | 15724 | 180372 | F |
| R99 | 32 | 65655 | 135328 | P |
| R100 | 32 | 81780 | 152700 | F |
| R101 | 32 | 135328 | 182796 | P |
| R102 | 31 | 9133 | 129671 | F |
| R103 | 31 | 16258 | 96069 | F |
| R104 | 31 | 16258 | 106342 | F |
| R105 | 31 | 81646 | 137529 | F |
| R106 | 31 | 86024 | 96069 | F |
| R107 | 31 | 86024 | 106342 | F |
| R108 | 31 | 129271 | 129671 | P |
| R109 | 31 | 129671 | 155307 | P |
| R110 | 31 | 5022 | 172934 | P |
| R111 | 31 | 12174 | 219265 | P |
| R112 | 31 | 29568 | 166077 | P |
| R113 | 31 | 106529 | 106565 | F |
| R114 | 31 | 116547 | 138378 | P |
| R115 | 31 | 116832 | 138751 | P |
| R116 | 31 | 116832 | 239939 | F |
| R117 | 30 | 165539 | 222336 | P |
| R118 | 30 | 18490 | 83676 | P |
| R119 | 30 | 18490 | 165150 | P |
| R120 | 30 | 45849 | 109471 | P |
| R121 | 30 | 59137 | 149925 | F |
| R122 | 30 | 85374 | 96069 | F |
| R123 | 30 | 85374 | 106342 | F |
| R124 | 30 | 96069 | 104583 | P |
| R125 | 30 | 104583 | 106342 | P |
| R126 | 30 | 149925 | 165178 | P |
| R127 | 30 | 149925 | 176278 | F |

*P represents palindromic repeats, F represents forward repeats.

**TABLE 8.** Comparison of dispersed repeats in *H. citrina* and five other monocotyledons.

| **Species** | **Total Number of Repeats** | **Total Length of Repeats (bp)** | **Genome Size (bp)** | **Proportion in Genome (%)** |
| --- | --- | --- | --- | --- |
| *Hemerocallis citrina* | 127 | 35,337 | 468,462 | 7.5 |
| *Asparagus officinalis* | 113 | 26,729 | 492,062 | 5.4 |
| *Allium cepa* | 194 | 12,155 | 316,363 | 3.8 |
| *Cocos nucifera* | 195 | 81,999 | 678,653 | 12.1 |
| *Spirodela polyrrhiza* | 38 | 3,129 | 228,493 | 1.4 |
| *Zea mays* | 232 | 60,754 | 569,630 | 10.7 |

**TABLE 9.** Distribution of PCGs in the *H. citrina* mitogenome.

| **Gene Name** | **Chromosome** | **Length** | **Start Codon** | **Stop Codon** | **Direction** |
| --- | --- | --- | --- | --- | --- |
| *atp1* | MZ726801 | 1,530 | ATG | TGA | R |
| *atp4* | MZ726802 | 585 | ATG | TAA | F |
| *atp6* | MZ726803 | 744 | ATG | TGA | R |
| *atp8* | MZ726801 | 843 | ATG | TAA | R |
| *atp8* | MZ726801 | 465 | ATG | TAA | F |
| *atp9* | MZ726801 | 261 | - | TGA | R |
| *atp9* | MZ726802 | 261 | - | TGA | R |
| *ccmB* | MZ726802 | 621 | ATG | TGA | F |
| *ccmC* | MZ726802 | 819 | ATG | TAG | F |
| *ccmFc* | MZ726802 | 1,347 | ATG | TAA | F |
| *ccmFn* | MZ726803 | 1,842 | ATG | TAG | R |
| *cob* | MZ726802 | 1,170 | ATG | TAA | F |
| *cox1* | MZ726801 | 1,584 | ATG | TAA | R |
| *cox1* | MZ726802 | 1,584 | ATG | TAA | R |
| *cox2* | MZ726802 | 819 | ATG | TGA | F |
| *cox3* | MZ726803 | 798 | ATG | TGA | R |
| *matR* | MZ726802 | 2,019 | ATG | TAA | F |
| *matR* | MZ726802 | 2,019 | ATG | TAA | R |
| *matR* | MZ726803 | 1,959 | ATG | TAA | F |
| *mttB* | MZ726803 | 759 | - | TAG | F |
| *nad1* | MZ726802-MZ726803 | 975 | ACG | TAA | F/R |
| *nad2* | MZ726802-MZ726803 | 1,467 | ATG | TAA | R |
| *nad3* | MZ726802 | 357 | ATG | TAA | F |
| *nad4* | MZ726803 | 1,488 | ATG | TGA | F |
| *nad4L* | MZ726802 | 303 | ACG | TAA | F |
| *nad5* | MZ726802-MZ726803 | 2,019 | ATG | TAA | F/R |
| *nad6* | MZ726803 | 696 | ATG | TGA | F |
| *nad7* | MZ726802 | 1,185 | ATG | TAG | F |
| *nad9* | MZ726802 | 573 | ATG | TAA | F |
| *rpl16* | MZ726802 | 426 | GTG | TAA | F |
| *rpl16* | MZ726803 | 426 | GTG | TAA | F |
| *rpl5* | MZ726802 | 588 | ATG | TAA | F |
| *rps1* | MZ726802 | 501 | ATG | TAA | F |
| *rps10* | MZ726802 | 336 | ACG | TGA | F |
| *rps10* | MZ726802 | 336 | ACG | TGA | R |
| *rps10* | MZ726803 | 336 | ACG | TGA | F |
| *rps12* | MZ726802 | 378 | ATG | TGA | F |
| *rps13* | MZ726803 | 351 | ATG | TGA | R |
| *rps14* | MZ726802 | 303 | ATG | TAG | F |
| *rps2* | MZ726802 | 696 | ATG | TAG | F |
| *rps3* | MZ726802 | 1,686 | ATG | TAG | F |
| *rps3* | MZ726803 | 1,686 | ATG | TAG | F |
| *rps4* | MZ726803 | 1,035 | ATG | TAA | F |
| *rps7* | MZ726803 | 351 | - | TAA | F |
| *sdh4* | MZ726802 | 255 | CTG | TGA | R |

## Supplementary Figures


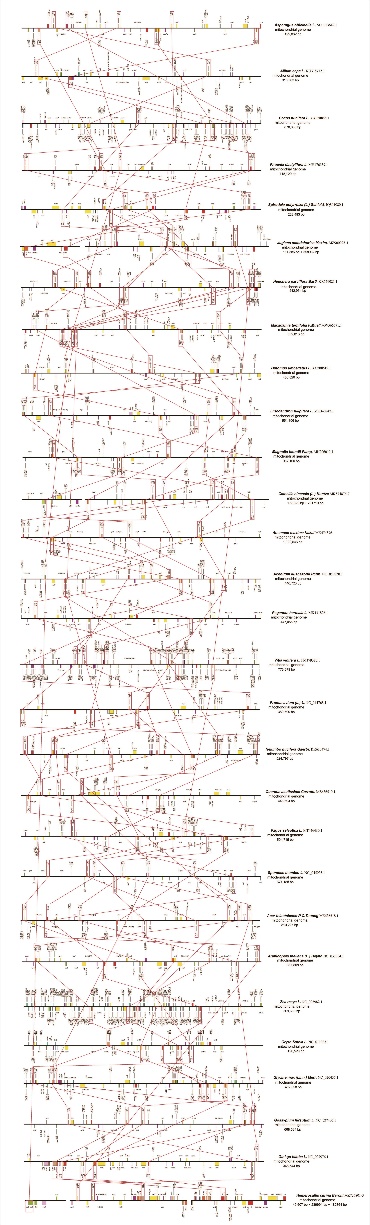


**FIGURE 1.** Identification of conservative gene clusters in the *H. citrina* mitogenome and 28 other higher plant mitogenomes.


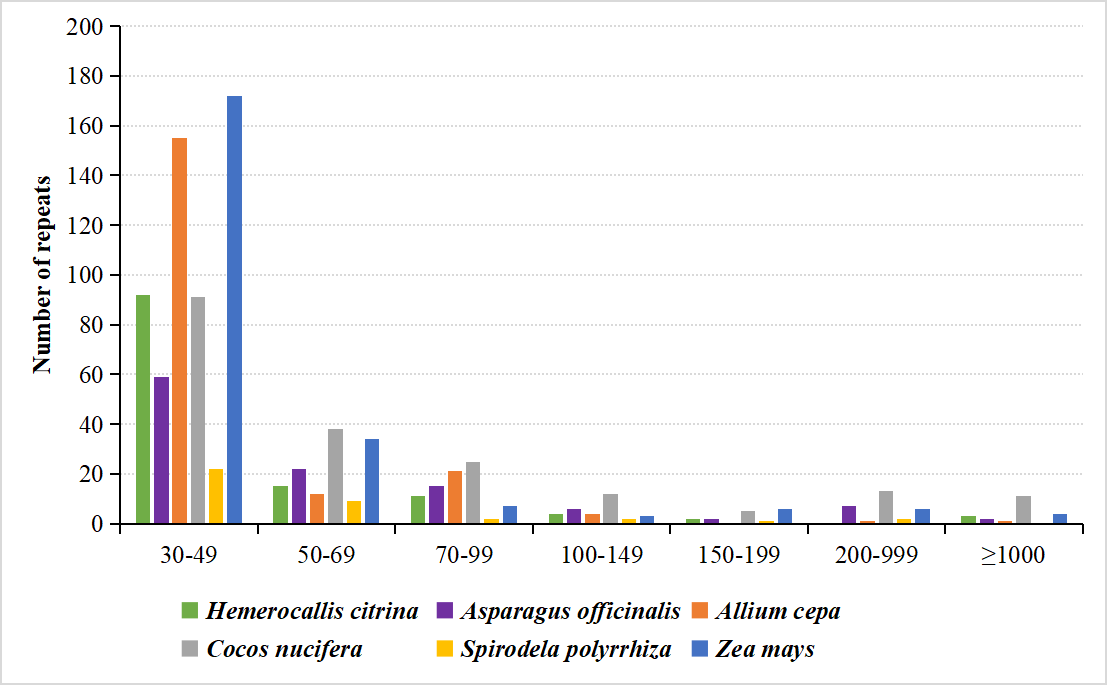


**FIGURE 2.** Dispersed repeats in *H. citrina* and five other monocotyledons.

**
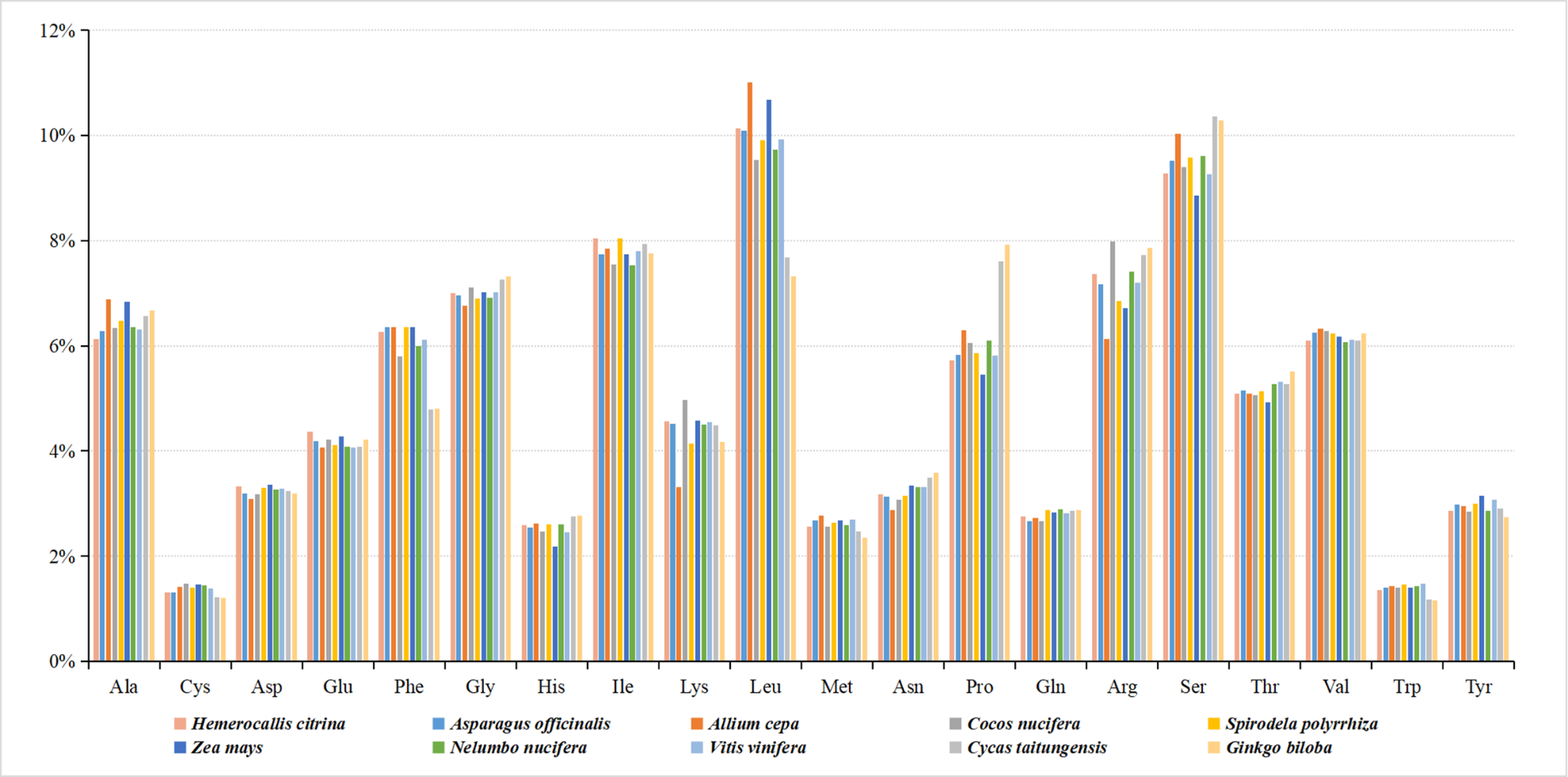
**

**FIGURE 3.** Codon usage pattern of *H. citrina* and nine other species. Amino acid residues are displayed on the X-axis. The Y-axis indicates the proportion of particular amino acid residues in the entire mt proteins.


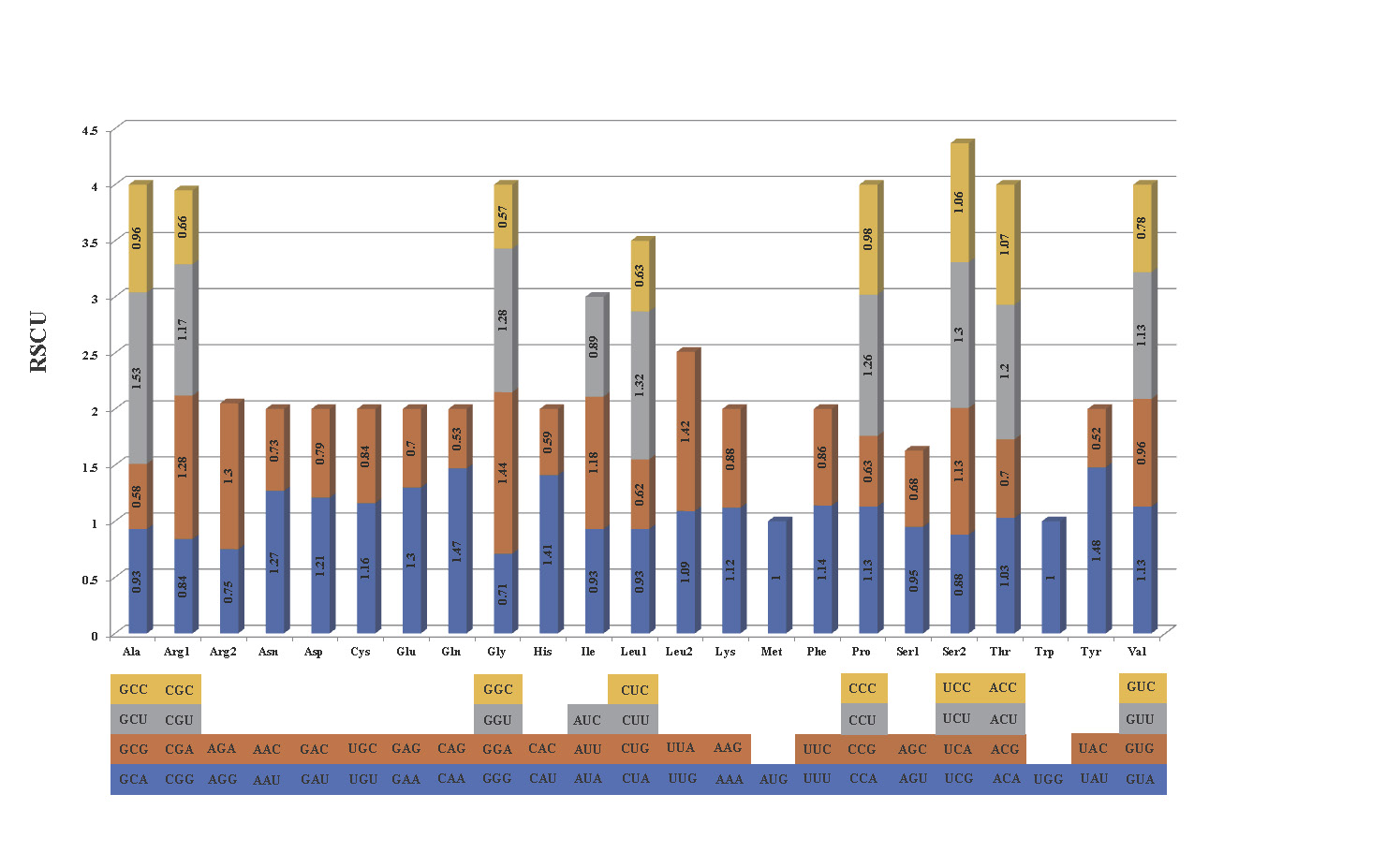


**FIGURE 4.** Determination of relative synonymous codon usage (RSCU) in *H. citrina*. The X-axis represents codon families, and the Y-axis represents the RSCU values for a particular codon.
